# Safety data on single application of emu and macadamia nut oil on human skin

**DOI:** 10.1016/j.dib.2017.10.026

**Published:** 2017-10-19

**Authors:** Tadayoshi Miyashita, Ryosuke Koizumi, Yoshimasa Sagane, Kazuhiro Minami, Minoru Ito, Toshihiro Watanabe, Koichi Niwa

**Affiliations:** a DHC Corporation, 2-7-1 Minami-Azabu, Minato-ku, Tokyo 106-8571, Japan; b NODAI Research Institute, 1-1-1 Sakuragaoka, Setagaya-ku, Tokyo 156-8502, Japan; c Department of Food and Cosmetic Science, Faculty of Bioindustry, Tokyo University of Agriculture, 196 Yasaka, Abashiri 099-2493, Japan

**Keywords:** Emu, Macadamia nut, Safety, Patch test

## Abstract

This data article provides the results of skin sensitization testing for emu and macadamia nut oil on 20 participants (ages 22–59 years old), including 3 men and 17 women. The test was carried out by performing a standard patch test using a Finn Chamber on Scanpor tape. The oils were applied to the participant's back using the tape and left in place for 24 h. After 1- and 24-h from removal of the tape, the reaction of the participant's skin was judged based on a scoring method recommended by Japanese Patch Test Research Group. Results are shown in table format.

**Specifications Table**

**Table:** 

Subject area	*Health science*
More specific subject area	*Cosmetic science*
Type of data	*Table*
How data was acquired	*Skin sensitization test on human participants*
Data format	*Raw*
Experimental factors	*Commercially available emu oil and macadamia nut oil were employed*
Experimental features	*Reaction of human skin was observed after application of the sample oils to the participant's back using a Finn Chamber on Scanpor tape (Smart Practice Dermatology, Phoenix, AZ, USA).*
Data source location	*Abashiri, Japan*
Data accessibility	*Data is within this article*

**Value of the data**

• The data presented offer a valuable and searchable resource showing the effects of emu oil and macadamia nut oil on human skin.
• The data presented here could be applied to ensure the safety of emu oil and macadamia nut oil on human skin.
• The data presented are available as reference to compare the effects of emu oil and macadamia nut oil with other oils.

## 1 Data

This data article contains results of skin sensitization testing for emu oil and macadamia nut oil on 20 participants, as shown in Table 1.

**Table 1:** Results of skin sensitization test. The reaction of the skin was observed 1- and 24-h later after removal of the sample tape.

Time after removal	1 h	24 h
Sample	Emu oil	
−	20	20
±	0	0
+	0	0
++	0	0
+++	0	0
++++	0	0
	Macadamia nut oil	
−	19	20
±	1	0
+	0	0
++	0	0
+++	0	0
++++	0	0
	White petrolatum	
−	20	20
±	0	0
+	0	0
++	0	0
+++	0	0
++++	0	0
	Physiological saline	
−	20	20
±	0	0
+	0	0
++	0	0
+++	0	0
++++	0	0
	Distilled water for injection	
−	20	20
±	0	0
+	0	0
++	0	0
+++	0	0
++++	0	0

## 2 Experimental design, materials, and methods

### 2.1 Design

Skin sensitization testing was performed by patch test on 20 healthy participants with emu oil and macadamia nut oil. For negative controls, white petrolatum, physiological saline, and distilled water for injection were employed. Data were collected between May 9th and 11th, 2017. The study was performed in accordance with the Helsinki Declaration and Japanese ethical guidelines for medical and health research involving human participants.

### 2.2 Materials

Commercially available emu oil and macadamia nut oil were supplied from Tokyo NODAI Bioindustry Co. Ltd. (Abashiri, Japan).

### 2.3 Participants

Twenty healthy participants were collected according to the inclusion and exclusion criteria shown in Supplementary Document 1. An informed consent document was obtained from each participant prior to the study.

### 2.4 Application of patch test

The materials were applied to participant's upper back using a patch test unit (Finn Chamber on Scanpor Tape; Smart Practice Dermatology, Phoenix, AZ, USA). After 24 h, the patches were removed. One hour later, the sites were photographed and evaluated. Twenty-four hours after the removal of the patches, the sites were again photographed and evaluated.

### 2.5 Evaluation of dermal response

Numerical equivalents recommended by the Japanese Patch Test Research Group [1] were adopted to measure the dermal response observed at the time of patch removal (Supplementary Table 1).
