# Supplementary material for: Safety data on single application of emu and macadamia nut oil on human skin
Source: Data Brief. 2017 Oct 19;15:720–3. doi: 10.1016/j.dib.2017.10.026 (PMC5671474; doi:10.1016/j.dib.2017.10.026)
Supplement: Supplementary file 1 — Supplementary material [file mmc1.docx]

**Conflict of interests:** None
