# Supplementary material for: Safety data on single application of emu and macadamia nut oil on human skin
Source: Data Brief. 2017 Oct 19;15:720–3. doi: 10.1016/j.dib.2017.10.026 (PMC5671474; doi:10.1016/j.dib.2017.10.026)
Supplement: Supplementary file 2 — Supplementary material [file mmc2.docx]

**Supplementary document 1**

Participant inclusion and exclusion criteria

Inclusion criteria

1. Generally healthy male or female individual between the ages of 20 and 65 years, inclusive
2. Able to understand the study and to read, understand, and sign an informed consent agreement

Exclusion criteria

1. Evidence of psoriasis and/or active atopic dermatitis
2. Skin disease at the study site which might interfere with the evaluation
3. History of drug allergy
4. Prone to allergic reaction, such as exanthema or urticaria
5. Use of any systemic or topical drugs or medication that would interfere with the study results
6. History of serious disease
7. History of drug or alcohol dependence
8. Pregnancy, lactation, or planning to be pregnant during the study time period
9. Use of drug(s) which would interfere with the study results within 2 weeks prior to the study
10. History of patch testing within 4 months prior to the study
11. Planning to participate in a human study during the study period
12. Participant who, in the opinion of the principle investigator, is inadequate for participation in the test.

**Supplementary Table 1**

Evaluation of patch test reaction

| Readings | Reactions |
| --- | --- |
| − | No reaction |
| ± | Slight erythema |
| + | Clear erythema |
| ++ | Erythema and papules or edema |
| +++ | Strong vesicular reaction |
| ++++ | Extreme bullous reaction |
